# Human papillomavirus infection as a prognostic marker for lung adenocarcinoma: a systematic review and meta-analysis

**DOI:** 10.18632/oncotarget.15671

**Published:** 2017-02-24

**Authors:** Lanwei Guo, Shuzheng Liu, Shaokai Zhang, Qiong Chen, Meng Zhang, Peiliang Quan, Xibin Sun

**Affiliations:** ^1^ Department of Cancer Epidemiology, Henan Office for Cancer Control and Research, The Affiliated Cancer Hospital of Zhengzhou University, Henan Cancer Hospital, Zhengzhou, China

**Keywords:** HPV, lung cancer, prognosis, meta-analysis

## Abstract

Although a number of studies have investigated the association between human papillomavirus (HPV) and lung cancer prognosis, the results remain inconsistent. We therefore conducted a meta-analysis of epidemiologic studies to address this issue. Searches of the MEDLINE and EMBASE electronic databases from their inception until June 30, 2016 yielded nine studies involving a total of 1,205 lung cancer cases that were used to conduct the meta-analysis. Study-specific risk estimates were pooled using a random-effects model. The pooled hazard ratio (HR) comparing HPV-positive to HPV-negative cancers 1.00 (95% confidence interval (CI): 0.78-1.28) was not significantly correlated with overall survival. However, lung adenocarcinoma patients with HPV infections exhibited a survival benefit compared to those without HPV infection (HR=0.69, 95% CI: 0.50-0.96). This meta-analysis suggests HPV infection is a prognostic marker in lung adenocarcinoma. To further elucidate the epidemiology and pathogenesis of HPV infections in lung cancer, future large prospective studies are encouraged to stratify survival analysis based on the pathological type and clinical stage of the cancer.

## INTRODUCTION

Globally, lung cancer is the most common cancer overall for several decades, with an estimated 1.8 million new cases in 2012 and also the most common cause of death from cancer with an estimated 1.6 million deaths [1]. The number of lung cancer deaths are expected to reach 2.9 million by the year 2035 [1].

Although cigarette smoking is a predominant factor for lung cancer incidence, 25% of lung cancer patients are nonsmokers. There are many other potential risk factors for lung cancer, such as occupational or environmental exposure to radon and asbestos, certain metals and air pollution, as well as infectious diseases [2, 3]. As reported, the high-risk oncogenic human papillomavirus (HPV) prevalence was 89.7% in cervical cancer [4], 29.5% in head and neck cancer [5] and 22.0% in lung cancer [6]. And HPV has been identified as a causal agent in a variety of human carcinomas, including cervix cancer and head and neck cancer [7–9]. It was not clear whether HPV was implicated in lung carcinogenesis.

One study reported that HPV-positive cervical cancer patients who receiving radiation therapy had significantly better survival [10]. Some retrospective clinical studies consistently proved that HPV-positive head and neck squamous cell carcinoma (HNSCC) patients had a better prognosis than those HPV-negative [11–14]. Lungs can be infected just like the oral cavity, tonsils, and pharynx, it is supposed that the histological similarities between the head and neck squamous epithelia and lung suggest a similar association and clinical characteristics. Although the prognostic value of the HPV status has been investigated in patients with lung cancer previously, the results have often been controversial.

Therefore, this systematic review and meta-analysis was conducted to clarify the association between HPV infection and overall survival (OS) in lung cancer patients.

## RESULTS

### Literature search

**Figure 1:**
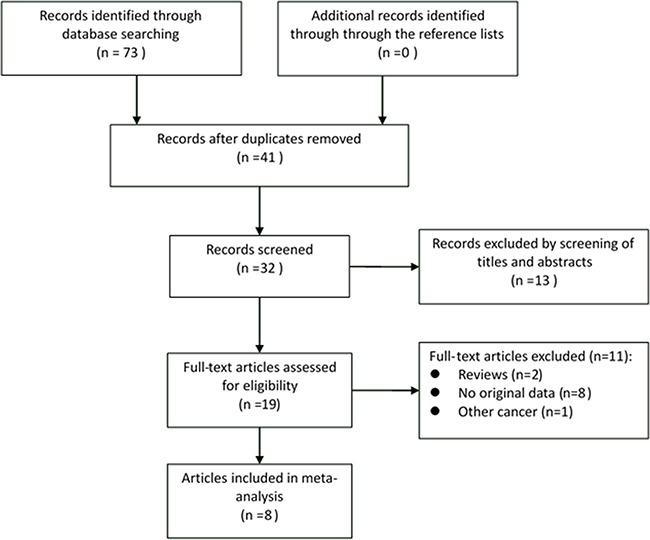
Flow diagram of systematic literature search

As shown in Figure 1, a total of 73 citations were generated, of which 32 were considered of potential value after screening titles and abstracts, and 19 articles were remained and retrieved for detailed evaluation after reading the full text. Eleven of these 19 articles were subsequently excluded for various reasons, including two reviews, eight that did not provide *HR*s and one that was not based on lung cancer. Finally, eight eligible articles were included in this systematic review and meta-analysis [15–22].

### Characteristics of the selected articles

Individual characteristics of the included 8 articles are summarized in Table 1. All included articles, satisfying all the five points proposed by MOOSE, had a very high quality. They were published from 2000 to 2014 and included a total of 1,205 lung cancer cases. Among these articles, five studies were conducted in China [16–18, 21, 22], two in Japan [19, 20] and one in Russia [15]. Of all the selected articles, three evaluated *HR*s [15, 16, 21], while in the other five articles [17–20, 22], *HR*s were absent, and needed to be calculated from the survival curves. Three articles did not give accurate data for follow-up [16, 19, 20]. The median follow-up period of other five articles ranged from 12.6 to 59.3 months.

**Table 1:** Characteristics of the included studies

First author	Year	Year of recruitment	Race	Stage	Pathological type	No. of Patients	HPV + ve*N* (%)	Age, y	Genotype(s)	DNA method	Median follow-up period (months)	Hazardratio
Iwamasa	2000	1993-1995	Asian	I-II	LSCC	41	25(61.0)	69.8(+)/70.8(-)	6,11,16,18	PCR	NA	SC
Miyagi	2001	1995-1997	Asian	I-II	LA+LSCC	120	41(34.2)	LA: 67.3(+)/66.9(-)LSCC: 66.3(+)/69.7(-)	6,11,16,18	PCR	NA	SC
Hsu	2009	2000-2006	Asian	I	NSCLC	171	17(9.9)	65.2 (37-83)	16,18	IMC	56.4	SC
Wu	2012	1998-2014	Asian	I-III	NSCLC	165	74(44.8)	NA	16,18	PCR	59.3	SC
Chen	2013	2002-2007	Asian	I-III	LC	319	91(28.5)	NA	16,18	PCR	57.6	SC
Anantharaman	2014	2007-2010	Caucasian	NA	LC	62	15(24.2)	62.1	21 types*	PCR	43.44	Report
Chen	2014	1993-2014	Asian	NA	NSCLC	117	62(53.0)	NA	16,18	PCR	NA	Report
Wang	2014	2003-2011	Asian	I-IV	LA	210	74(35.2)	69.5	16,18	PCR	12.6	Report

Abbreviations: HPV + ve, human papillomavirus positive; LSCC, lung squamous cell carcinoma; LA, lung adenocarcinoma; NSCLC, non-small cell lung cancer; LC, lung cancer; PCR, polymerase chain reaction; IMC, immunohistochemistry; SC, survival curve; NA, not available.

* Including 19 high-risk (HPV-16, 18, 26, 31, 33, 35, 39, 45, 51, 52, 53, 56, 58, 59, 66, 68a, 68b, 70, 73, 82) and 2 low-risk types (HPV-6, 11).

### Results of the meta-analysis

**Figure 2:**
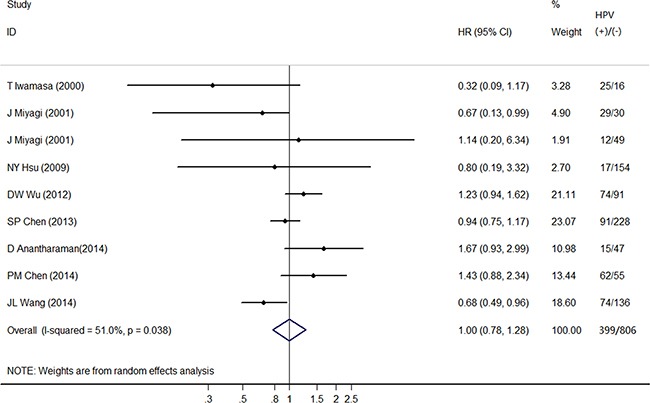
Forest plot comparing HPV-positive to HPV-negative lung cancer patients and overall survival

One included article [20] reported the *HR*s of lung adenocarcinoma and lung squamous cell carcinoma, respectively, and hence it was treated as two studies. Among the nine studies included, five [17–21] showed negative association comparing HPV-positive to HPV-negative cancers, one [20] showed statistical significance and the other four [15, 16, 20, 22] showed positive associations without statistical significance. The heterogeneity test indicated moderate degree of heterogeneity among included studies (Q-test *P*_heterogeneity_ =0.038, *I*^2^=51.0%) and therefore, random effects model was used to obtain the pooled *HR*. The pooled *HR* of HPV-positive to HPV-negative cancers was 1.00 (95% *CI*: 0.78-1.28) according to the nine individual effect estimates, suggesting no significant correlation with OS (Figure 2).

#### Subgroup analyses

Table 2 presents detailed results of subgroup analyses. The associations of HPV status and OS in lung cancer patients did not differ by study type, race, number of patients, detection method, HPV type, max follow-up time, case diagnosis method and hazard ratio. HPV status was significantly associated with improved OS for lung adenocarcinoma patients (*HR*=0.69, 95% *CI*: 0.50-0.96), but was not significantly associated with OS for lung squamous cell carcinoma patients (*HR*=0.50, 95% *CI*: 0.23-1.12), non-small cell lung cancer patients (*HR*=0.92, 95% *CI*: 0.65-1.31) and lung cancer patients (*HR*=1.17, 95% *CI*: 0.68-2.03). When cancer cases were stratified by treatment method, HPV status was not significantly associated with improved OS for surgery only (*HR*=0.97, 95% *CI*: 0.73-1.28), but was significantly associated with better OS for other treatment methods (*HR*=0.72, 95% *CI*: 0.58-0.89). In short, the estimated heterogeneity remained for the included studies although it decreased to some extent.

**Table 2:** Results of subgroup analyses

Group	No. of study	*HR* (95% *CI*)	Heterogeneity test	
			*P* for *Q* test	*I*^2^, % †
All	9	1.00 (0.78-1.28)	0.038	51.0
Study type				
Prospective	8	0.94 (0.74-1.21)	0.068	46.9
Race				
Asian	8	0.94 (0.74-1.21)	0.068	46.9
Number of patients				
<100	4	0.86 (0.43-1.72)	0.134	46.2
≥100	5	1.01 (0.76-1.34)	0.031	62.3
Pathological type				
LC	2	1.17 (0.68-2.03)	0.071	69.2
NSCLC	7	0.92 (0.65-1.31)	0.042	54.1
LSCC	2	0.50 (0.23-1.12)	0.376	0.0
LA	2	0.69 (0.50-0.96)	0.565	0.0
Detection method				
PCR	8	1.01 (0.78-1.31)	0.023	56.9
HPV type				
16,18	6	1.06 (0.82-1.38)	0.028	60.2
HR/LR-HPV	4	0.88 (0.40-1.91)	0.092	53.4
Max follow-up				
<5 years	2	0.77 (0.32-1.84)	0.603	0.0
≥5 years	6	0.96 (0.71-1.29)	0.019	63.1
Treatment method				
Surgery only	6	0.97 (0.73-1.28)	0.308	16.4
Others*	4	0.72 (0.58-0.89)	0.641	0.0
Case diagnosis method				
Pathology reports	8	1.10 (0.87-1.39)	0.174	31.8
Hazard ratio				
Reported	3	1.14 (0.62-2.07)	0.007	80.1
Estimated	6	0.99 (0.79-1.25)	0.283	20.0

Abbreviation: *HR*, hazard ratio; *CI*, confidence intervals; LC, lung cancer; NSCLC, non-small cell lung cancer; LSCC, lung squamous cell carcinoma; LA, lung adenocarcinoma; PCR, polymerase chain reaction; IMC, immunohistochemistry; HR, high-risk; LR, low risk;

† *I*^2^ is interpreted as the proportion of total variation across studies that are due to heterogeneity rather than chance;

*Including chemotherapy, radiotherapy, tyrosine kinase inhibitors and multiple therapies.

### Influence analysis of individual studies

**Figure 3:**
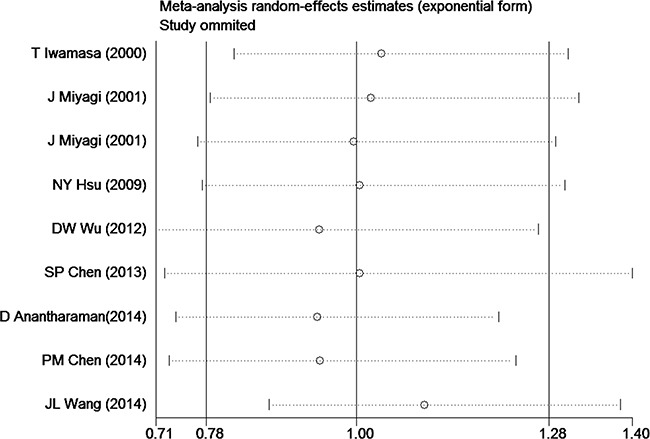
Influence analyses for omitting individual study on the summary *HR* for overall survival

To address the potential bias due to the quality of included studies, we performed the sensitivity analysis by calculating pooled *HR*s again by omitting one study at a time. And related results were showed in Figure 3. The pooled *HR*s comparing HPV-positive to HPV-negative cancers ranged from 0.94 (95% *CI*: 0.74-1.21) to 1.10 (95% *CI*: 0.87-1.39), which indicated that each single study didn't influence the stability of pooled *HR* estimate.

### Publication bias

**Figure 4:**
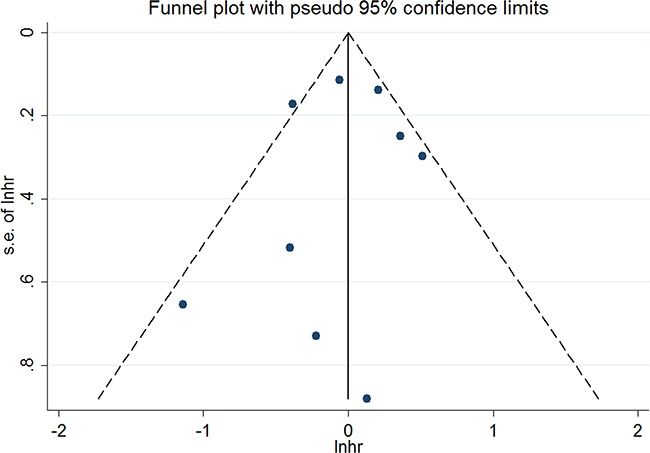
Funnel plots for publication bias of overall survival

Both the non-significant *P* values of Begg's test (1.000), Eegg's test (0.760), and the near-symmetric funnel plot demonstrated that there was no publication bias (Figure 4).

## DISCUSSION

As we know, this systematic review has its first-ever try on investigating overall survival of HPV-related lung cancers. The pooled effect estimates showed that lung adenocarcinoma patients with HPV infections survived better than those without.

The association between HPV infection and the occurrence of lung cancer was firstly reported by Syrjänen in 1979 [23], and several studies further explored impact of HPV infection on lung cancer prognosis. However, the conclusions of these studies were inconsistent. One previous study reported that HPV-positive HNSCCs was associated with a 54% reduction in overall mortality, in comparison to HPV-unrelated HNSCCs [24]. However, no association was found between HPV status and lung cancer prognosis in this meta-analysis. Among the included studies, only Wang *et al*. [21] demonstrated that patients with HPV-positive lung adenocarcinoma had a superior prognosis than patients with HPV-negative ones, with a 32% reduction in overall mortality. However, it is unclear about the biologic basis for the improved survival among the HPV positive patients and further study is needed.

Although limitations existed due to observational nature, it deserved to note some findings from subgroup-analysis. HPV infection was associated with improved survival for lung adenocarcinoma patients, but was not associated with improved survival for lung squamous cell carcinoma patients. Besides, when stratified by treatment method, HPV status was not significantly associated with improved OS for surgery only patients, but significantly associated with improved OS for patients with other treatment methods, such as chemotherapy, radiotherapy, tyrosine kinase inhibitors and multiple therapies. Further studies on the difference in survival between HPV status and different pathological types of lung cancer with different treatment methods were encouraged.

Clinical stage at diagnosis is the most important prognostic factor for lung cancer [25]. It's also a pre-requisite for identifying lung cancer patients as candidates for chemo-radiotherapy prior to surgery. For lung adenocarcinoma, one included study [21] showed HPV-positive stage I-IV patients having favorable survival, but HPV-positive patients stage I-II showed poorer survival in another study [20]. For LSCC, two studies [19, 20] showed HPV-positive stage I-II patients having favorable survival. For NSCLC (except lung adenocarcinoma and LSCC), one included study [18] showed HPV-positive stage I NSCLC patients having favorable survival, but HPV-positive patients stage I-III or I-IV showed poorer survival in two other studies [16, 22]. However, only three studies reported the adjusted *HR*s, one [16] of which reported the adjusted *HR*s for clinical stage. The favorable prognosis for HPV-infected lung adenocarcinoma or NSCLC patients compared with HPV-non-infected patients could be due to different tumor stages in the patients. So, it is recommended to do detailed survival analysis by different clinical stages of lung cancer for future studies.

Obviously, this systematic review and meta-analysis has several strengths. First, it is the first time to explore survival differences in different HPV status among lung cancers to date, and this comprehensive review is the most methodologically robust. Second, rigorous inclusion/exclusion criterion and advanced meta-analysis of *HR* for survival were applied. Moreover, a variety of subgroup analyses were conducted, which means a minimized potential confounders. In addition, no publication bias and the robust results of sensitivity analysis indicated the reliability of our study.

Limitations of this meta-analysis should be considered. First, potential bias could not be completely excluded considering that different HPV DNA detection methods were used in the included studies, and the estimates of HPV infection might be influenced largely due to the difference of the sensitivity and accuracy of the detection methods. Second, though random-effects model meta-analysis was used whenever significant heterogeneity was noted and sensitivity with subgroup analyses were performed to figure out potential sources of heterogeneity, significant heterogeneity was observed. Third, only those articles published in English were included here, which may have introduced language bias as well. Finally, only one third included studies reported the adjusted *HR*s, which may exclude other potential prognostic factors such as smoking.

In summary, no association was observed between HPV infection and lung cancer survival. However, HPV infection may be a prognostic marker in lung adenocarcinoma, which suggests that assessment of HPV infection in clinical practice might help to determine the relevant treatment regimen. To further elucidate the epidemiology and pathogenesis of HPV infections in lung cancer, future large prospective studies are encouraged to stratify survival analysis based on the pathological type and clinical stage of the cancer.

## MATERIALS AND METHODS

### Literature search strategy

A systematic search up to 30 Jun 2016 was conducted in MEDLINE (via PubMed) and Excerpta Medica database (EMBASE) to identify relevant articles. Search terms included ‘‘human papillomavirus OR HPV”, ‘‘lung cancer OR lung neoplasms OR lung carcinoma’’ combined with “prognosis OR prognostic OR survival”. Additional relevant references cited in retrieved articles were also evaluated.

### Inclusion and exclusion criteria

All papers were reviewed by two authors (S.Z. and Q.C.) independently. Uncertainties and discrepancies were resolved by consensus after discussing with a senior researcher (P.Q.). All studies included in the final meta-analysis satisfied the following criteria: (a) patients were pathologically diagnosed as lung cancer; (b) lung cancer OS as the outcome of interest; (c) reported *HR* estimates with their corresponding 95% *CI* (or sufficient data to calculate of these effect measure), and (d) English articles. If the study was reported in duplication, the one published earlier or provided more detailed information was included. Review articles and editorials were included if they contained original data. Abstracts were excluded.

### Quality assessment

According to a critical review checklist of the Dutch Cochrane Centre proposed by MOOSE, we strictly assessed the quality of all the studies included [26]. (i) clear definition of study population and origin of country; (ii) clear definition of study design; (iii) clear definition of outcome assessment; (iv) clear definition of HPV detection method and (v) sufficient period of follow-up. Otherwise, we would exclude the studies in order to ensure the quality of the meta-analysis.

### Data extraction

Two of the authors (S.L. and S.Z.) performed the data extraction from each article and discrepancies were resolved by consensus. For studies meeting our inclusion criteria, a standardized data extraction form was used to extract the following data: the first author's name, year of publication, country of origin, study design, period of enrollment, the length of follow-up, characteristics of the studied population (sample size, age, stage of disease and treatment method), HPV detection methods, and *HR* estimates for OS with corresponding 95% *CI*s. When data for *HR* was not available, we extracted the total numbers of observed deaths and the numbers of patients in each group to calculate *HR* [27]. Data were extracted by Engauge Digitizer version 4.1 (http://digitizer.sourceforge.net/) from the graphical survival plots when data were only available as Kaplan-Meier curves [28], then the estimation of the *HR* was performed by the described method [27].

### Statistical analysis

The *HR* with 95% *CI* was used to compute the pooled HPV infections and the OS in lung cancer patients. A random-effect model was used to pool the data, based on the DerSimonian and Laird method [29].

Cochrane *Q* test *(P* < 0.10 indicated a high level of statistical heterogeneity) and *I*^2^ (values of 25%, 50% and 75% corresponding to low, moderate and high degrees of heterogeneity, respectively) was used to assess the heterogeneity between eligible studies, which test total variation across studies that was attributable to heterogeneity rather than to chance [30]. Subgroup analyses for HPV infections and the OS in lung cancer patients were subsequently carried out according to the study type, race, number of patients, pathological type, detection method, HPV type, max follow-up time, treatment method, case diagnosis method and hazard ratio. Sensitivity analysis was also conducted to assess the influence of each individual study on the strength and stability of the meta-analytic results. Each time, one study in the meta-analysis was excluded to show that study's impact on the combined effect size. Funnel plot and Begg adjusted rank correlation test for funnel plot asymmetry were performed to test any existing publication bias.

All statistical analyses were performed using STATA version 12 for Windows (StataCorp LP, College Station, TX, USA). A two-tailed *P*<0.05 was considered statistically significant.
